# Major Infection Events Over 5 Years: How Is Media Coverage Influencing Online Information Needs of Health Care Professionals and the Public?

**DOI:** 10.2196/jmir.2146

**Published:** 2013-07-15

**Authors:** Patty Kostkova, David Fowler, Sue Wiseman, Julius R Weinberg

**Affiliations:** ^1^Department of Computer ScienceNational Resource for Infection Control (NRIC)University College LondonLondonUnited Kingdom; ^2^National Resource for Infection Control (NRIC)LondonUnited Kingdom; ^3^Vice Chancellor's OfficeKingston University LondonLondonUnited Kingdom

**Keywords:** information seeking behavior, weblogs analysis, online information needs, data mining, infectious outbreaks

## Abstract

**Background:**

The last decade witnessed turbulent events in public health. Emerging infections, increase of antimicrobial resistance, deliberately released threats and ongoing battles with common illnesses were amplified by the spread of disease through increased international travel. The Internet has dramatically changed the availability of information about outbreaks; however, little research has been done in comparing the online behavior of public and professionals around the same events and the effect of media coverage of outbreaks on information needs.

**Objective:**

To investigate professional and public online information needs around major infection outbreaks and correlate these with media coverage. Questions include (1) How do health care professionals’ online needs for public health and infection control information differ from those of the public?, (2) Does dramatic media coverage of outbreaks contribute to the information needs among the public?, and (3) How do incidents of diseases and major policy events relate to the information needs of professionals?

**Methods:**

We used three longitudinal time-based datasets from mid-2006 until end of 2010: (1) a unique record of professional online behavior on UK infection portals: National electronic Library of Infection and National Resource of Infection Control (NeLI/NRIC), (2) equivalent public online information needs (Google Trends), and (3) relevant media coverage (LexisNexis). Analysis of NeLI/NRIC logs identified the highest interest around six major infectious diseases: *Clostridium difficile* (*C difficile*)/Methicillin-resistant *Staphylococcus aureus* (MRSA), tuberculosis, meningitis, norovirus, and influenza. After pre-processing, the datasets were analyzed and triangulated with each other.

**Results:**

Public information needs were more static, following the actual disease occurrence less than those of professionals, whose needs increase with public health events (eg, MRSA/*C difficile*) and the release of major national policies or important documents. Media coverage of events resulted in major public interest (eg, the 2007/2008 UK outbreak of *C difficile*/MRSA). An exception was norovirus, showing a seasonal pattern for both public and professionals, which matched the periodic disease occurrence. Meningitis was a clear example of a disease with heightened media coverage tending to focus on individual and celebrity cases. Influenza was a major concern during the 2009 H1N1 outbreak creating massive public interest in line with the spring and autumn peaks in cases; although in autumn 2009, there was no corresponding increase in media coverage. Online resources play an increasing role in fulfilling professionals’ and public information needs.

**Conclusions:**

Significant factors related to a surge of professional interest around a disease were typically key publications and major policy changes. Public interests seem more static and correlate with media influence but to a lesser extent than expected. The only exception was norovirus, exhibiting online public and professional interest correlating with seasonal occurrences of the disease. Public health agencies with responsibility for risk communication of public health events, in particular during outbreaks and emergencies, need to collaborate with media in order to ensure the coverage is high quality and evidence-based, while professionals’ information needs remain mainly fulfilled by online open access to key resources.

## Introduction

### Background

There is a large amount of medical information available on the Internet, ranging from specialist databases and indexed collections of articles for health care professionals to less technical information sites for the general public. It is estimated that around 80% of the general public and a comparable proportion of medical professionals access information via the Internet [1]. In this paper, we examine the search behavior of visitors to a specialist medical online portal (in the domain of infectious diseases and infection prevention control) and the search behavior of the wider public using a search engine. We also consider the possible influence of media reporting of disease outbreaks on these behaviors.

The last decade witnessed turbulent events in the domain of infectious diseases and public health. New and emerging infections, such as Severe Acute Respiratory Syndrome (SARS), deliberately released threats (eg, anthrax), and ongoing battles with common illnesses, such as influenza, tuberculosis (TB), Healthcare Associated Infections (HAI), and the A/H1N1 swine flu pandemic outbreak of 2009 were amplified by the spread of disease through increased speed and volume of international travel. It is more important than ever to ensure that health care professionals and members of the public are well informed and kept up to date with the latest public health developments, government advice, and rapid risk communications. However, in addition to official health authorities’ communications, in the Internet era professionals and the public increasingly use online resources to meet their information needs and seek up-to-date evidence. Also, media coverage of infection outbreaks, public health issues, and media-mediated risk advice is increasingly influencing public perceptions and often distorting health critical information [2].

### Health-Related Information Seeking Behavior of Professionals and Public

As of the end of December 2009, there were an estimated 1.8 billion Internet users worldwide. In Europe, 53% of the population use the Internet, which rises to 77% in the United Kingdom (with 69% having a broadband connection) (values are from surveys quoted in Higgins et al [1]). More recent results [3] indicate that 56% of the population in the European Union use the Internet daily, with 68% using the Internet every week.

Various surveys ([4-5]) quoted by Higgins et al [1] indicate that 8 out of 10 Internet users in the United States use the Internet to access health information and that the corresponding number for Europe was 7 out of 10 (according to a 2007 study by Andreassen et al [6]). A study by Seybert in 2011 [3] found that 54% of EU Internet users used the Internet to look for health-related information (lower than the 71% mentioned by Andreassen et al). This difference might be explained by differences in sampling and the wording of questions (see [7] for a discussion on this subject). Overall, it seems reasonable to expect a continued increase in the proportion of Internet usage by the general public, as well as the proportion of those users seeking online health information.

In addition to the increased use of online resources by members of the public to manage their personal health and better understand their conditions, in recent years the online health information environment has become mobile, with 17% of cell phone users having used their phones to look up health information and 9% using software applications on their phones that help them track or manage their health [8].

While these studies provide cumulative data on Internet usage, it is also essential to investigate users’ search and online behavior to understand their online information needs and how these are fulfilled technically as well as in the context of site usability [9]. Furthermore, do members of the public access medical information online for the same reasons as health care professionals and does their search behavior differ?

A number of studies have investigated health care professionals’ online information seeking behavior. Younger gives a survey of studies comparing the search behavior of doctors and nurses [10]. It was difficult to compare individual studies due to the lack of harmonization of design and terminology, but the main conclusion was that many barriers exist for health care professionals, including lack of time and resources. There are also social barriers for professionals to use computers in the health care environment [11]. Alghanim [12] examines the information seeking behavior of primary health care physicians in Saudi Arabia, with one finding showing that around 50% of rural physicians used online databases and general websites to find information, rising to over 70% for urban physicians, with the difference presumably due to the lack of availability of these resources in rural areas. O’Keeffe et al [13] surveyed the information seeking behavior of a variety of health care personnel at two medical establishments in northern California (however the study does not distinguish exactly between online and offline information).

Public health and infection is one of the most varied domains of medicine, subject to rapid changes, disease outbreaks, and control measures involving the general public at regional, national, and international scales. As we run a specialist online digital library for infection and public health professionals and have a unique longitudinal online search dataset, we will focus on the information needs of the public and professionals regarding infection. It is not easy to say what drives the behavior of the public to seek information on particular infectious diseases. An actual outbreak of the disease could be a factor, but the knowledge of the outbreak will usually be obtained via mass media. The media’s reporting of disease outbreaks may be exaggerated due to certain needs, such as a need for a human interest angle [14].

### Effects of Media Coverage on Information Seeking Behavior

Media coverage of health-related news stories can influence the decisions and behavior of policy makers and the public [2]. For example, some parents refused to have their children vaccinated with the combined MMR (measles, mumps, and rubella) vaccine after intense media coverage of a single paper (later discredited [15]) linking the MMR vaccine to autism. Media coverage can be distorted, giving extra attention to stories about health concerns that have little real impact, while largely ignoring those (such as smoking, obesity, and alcohol) that cause much more harm [2].

Media coverage can also help to limit an outbreak, by causing individuals who are susceptible to the disease to isolate themselves from infected individuals [16]. Finally, the media coverage of a disease may be heightened even when there is no outbreak at the time. A good example is the reporting of the findings of an inquiry into an outbreak (eg, the coverage in October 2007 of *Clostridium difficile* (*C diff*), concerning a report into a prolonged outbreak between April 2004 and September 2006).

The 2009 swine flu outbreak was a health event covered extensively by the mass media and with an impact investigated by a number of research studies. Hilton and Hunt examined UK newspaper coverage of the 2009-10 swine flu (A/H1N1) outbreak [17]. They found that there was “immense” coverage in the spring and summer of 2009, when there was most uncertainty about the future impact on the United Kingdom. Later, in the autumn of 2009, there were few news articles, despite a second peak in the number of swine flu cases. Also, public information needs changed as members of public were overwhelmed by the information in the spring of 2009 but were less interested in the second half of 2009 [18].

Therefore, in this study we will investigate the following questions:

1. How do health care professionals’ online search needs around infection differ from the needs of the public?
2. Does media coverage contribute to the information needs among the public for infection?
3. How are incidents of a disease and major policy events related to the information needs of professionals?

##  Methods

We used 3 time-based datasets that were selected to cover the levels of interest in various infectious diseases and organisms. The datasets are intended to give a good representation of the search interests of health care professionals and the public and also the level of media coverage of each topic. We were not attempting in this study to prove causal relationships between the highly interrelated worlds of public, professionals, and media coverage, but rather to use a triangulation method [19] to examine the 3 related datasets and seek to make inferences about possible causal relationships between them.

###  Datasets

The 3 time-based datasets are:

1. The levels of user activity for various infection topics in the NeLI/NRIC specialist online digital library, run by City eHealth Research Centre (CeRC), City University, London. This unique dataset reflects the levels of interest in various topics by health care professionals.
2. The search statistics for the same infection topics from Google Trends [20]. This dataset reflects the levels of interest in the topics by the general public who seek health care information online on Google.
3. The numbers of news articles retrieved from the LexisNexis database, concerning the same topics as were used for the other datasets. This dataset represents the media coverage of the topics. Our search was restricted to English language coverage, but this includes major world newspapers in English.

We were interested in trends in the levels of activity in these datasets (whether activity was above or below the average level, and by how much, and whether activity was rising or falling over the long term or showing sudden peaks) and any correlations between the datasets.

The datasets are described in more detail in the following sections. As our primary interest was professional needs and their correlations, the most reliable results were ensured by selecting the diseases and conditions that had the highest activity levels on the NeLI/NRIC sites.

Table 1 gives the average weekly and peak NeLI/NRIC category accesses for various diseases or organisms, arranged in descending order. Unsurprisingly, as the user base of NeLI/NRIC is predominantly infection control professionals and the government nationally has increasingly focused on targets to reduce the top two infections listed below (Table 1), *C difficile* and multi-resistant *Staphylococcus aureus* (MRSA) lead the table followed by tuberculosis, meningitis, norovirus, and influenza.

**Table 1:** The average and peak weekly accesses for various diseases/organisms in NeLI/NRIC.

Disease/organism	Average weekly accesses	Peak weekly accesses
*C difficile* + MRSA	43.3	N/A
*C difficile*	23.9	72
MRSA	19.4	54
Tuberculosis	13.9	51
Meningitis	13.2	133
Norovirus	6.3	74
Influenza	2.6	21
SARS	1.4	34

In our analysis, we decided to combine the results for *C difficile* and MRSA. This was because (1) they are related topics (health care associated infections), (2) they are often mentioned together in media articles, and (3) public searches for “*Clostridium difficile*” were very few in comparison to searches for “MRSA”. This could possibly be due to the difficulty of spelling “*Clostridium difficile*” compared to “MRSA”. For this reason, we also looked at public searches for “superbug” because this lay term was frequently used in media and covers all HAIs. Another possibility is that the UK government targeted MRSA reduction first and only much later targeted *C difficile.*

The timeframe of the study was from week 31 (end of July) 2006 until the end of 2010, which is the period for which we have NeLI/NRIC data. The other datasets (Google Trends data and LexisNexis news article data) also cover this period.

####  Dataset 1: Professional Information User Needs—the NeLI/NRIC Portal Dataset

It is hard to determine the information needs of health care professionals. While surveys of behavior have been performed, the NeLI/NRIC server logs contain an invaluable record of actual search behavior in the domain of infection over several years.

**Figure 1:**
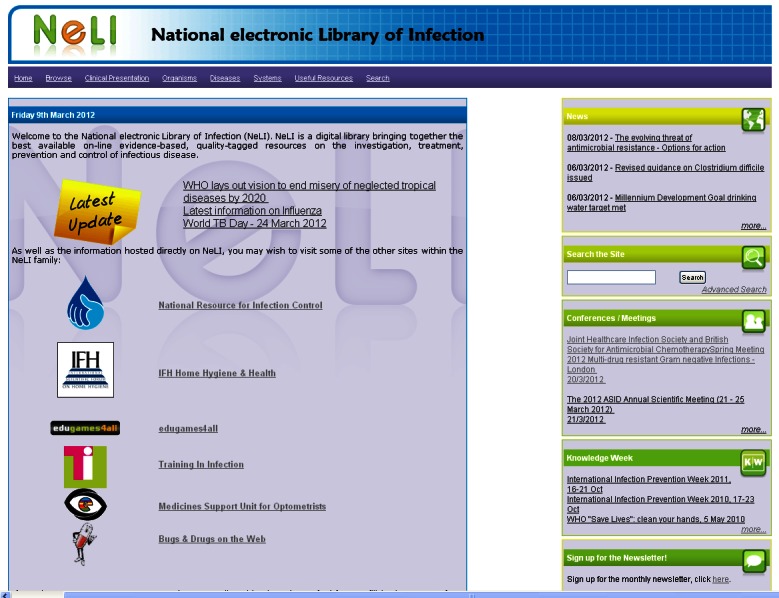
The National electronic Library of Infection (NeLI; information can be found by using drop down menus, left, or the search box, right).

Initially the specialist Library of Infection [21], part of the National electronic Library of Health (later NHS Evidence), The National electronic Library of Infection (NeLI) [22] is an online digital library created in 2000 at CeRC, with the aim of bringing together the best available evidence-based resources on the investigation, treatment, prevention, and control of infectious disease [23] (see Figure 1). Under the NeLI umbrella, several projects were developed using the same model, the largest of which is the National Resource for Infection Control (NRIC)[24], which was set up in May 2005 and specializes in resources on infection control and prevention. In the rest of the paper, we consider NeLI and NRIC together.

In addition to providing up to date evidence-based resources and stating the level of evidence of each resource (RCT, Meta-analysis, etc), a key benefit of NeLI/NRIC is that *Reviewer's Assessments* (RAs) are attached to documents within the library. These are written by professionals in the field and provide a short summary of what the document is about, highlighting any contradictory studies, potential bias, or conflicts of interest. Each review is signed by the reviewer and may be commented on by registered users. Documents can be found by searches (either simple keyword search or more complicated searches with Boolean operators) or by using a navigation structure based on a taxonomy of the domain developed with domain experts. Documents are organized in a two-level taxonomy that is also used for document indexing by domain experts and then further subdivided until documents about specific presentations, organisms, and diseases are found. In NeLI, the highest level categories are (1) Clinical Presentation, (2) Organisms, (3) Diseases, and (4) Systems, while in NRIC, the top level consists of (1) Settings, (2) Clinical Practice, (3) Transmission, (4) Diseases/Organisms, and (5) Policy/Guidance.

The source of professional health care interest data for our study is the Web traffic logs for NeLI/NRIC that have been automatically recorded since 2005. (Logs were recorded between 2001 and 2005 for NeLI, but as the site architecture changed in 2005, detailed comparisons between the logs for time periods before and after the change are not really possible).The Web server keeps a log of all Web accesses, and this record has been preserved since 2005.

**Figure 2:**
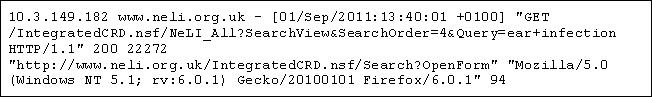
A sample log entry for a Web access to NeLI.

**Figure 3:**
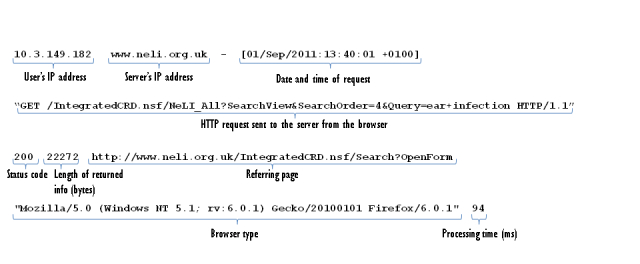
A sample log entry for a Web access to NeLI, annotated with the meanings of the available fields.

Each log entry contains details of an HTTP request sent from a Web browser to the Web server, including the date of each request, the IP address of the visitor, the page requested, and other data. Figure 2 shows a typical entry, and Figure 3 shows the same entry with explanations of the fields.

We can only observe visitors’ interactions with the NeLI/NRIC sites, with the notable exception that we can often determine which previous page they browsed before arriving at NeLI/NRIC (the *referring page*) that provides valuable information about user navigation behavior and successful promotion of the online resource.

##### NeLI/NRIC Users

**Figure 4:**
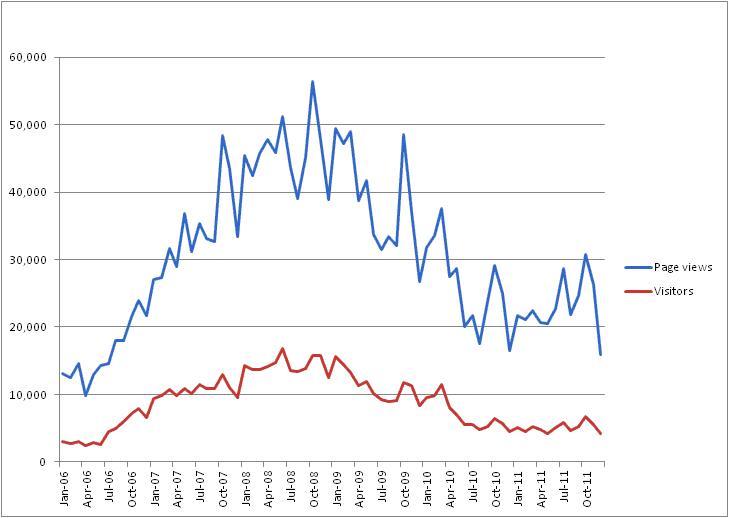
The numbers of visitors and page views for NeLI/NRIC between 2006 and 2011.

The NeLI/NRIC portals are aimed at health care professionals with interests in infection. Initially part of the NHS-led project, they were also promoted through the Health Protection Agency (HPA), the national public health agency in the United Kingdom. The site had over 5000 unique users per month in 2011 and between 20,000 and 30,000 page views (Figure 4).

From 2006 to 2008, NeLI/NRIC was heavily promoted at conferences and at other events, seemingly leading to an increase in visitor numbers. More recently, due to lack of resources, the site has been kept up to date, but promotional activity has lessened, resulting in a decline in site activity, clearly visible in the graph.

NeLI/NRIC is visited most frequently by users in the United Kingdom and the United States, and English speaking countries. However, despite the content being in English only and forming part of a national library, there is a growing number of users from countries such as India, Germany, and China, indicating a global need for such an evidence-based open access portal (see Supplementary Figure 1 in Multimedia Appendix 1).

All the content of the evidence-based library is in the public domain and free to use. In order to improve accessibility and usability no registration is required to access the content although users can subscribe to receive a monthly electronic newsletter that highlights the latest resources and upcoming events and conferences. Users can join the subscription list either personally at a conference, at a study day where NeLI/NRIC is presented, or online at a dedicated subscription page. The subscription database holds details for over 3500 NeLI/NRIC users. Subscribers listed in the database can provide their professions and specialities. Although the primary interest in subscribing to the site is “infection”, in order to better understand the professional backgrounds of NeLI/NRIC users an analysis of these was performed, and the breakdowns of professions and specialities are detailed in Supplementary Figures 2 and 3 in Multimedia Appendix 1.

#### Dataset 2: Public Information Needs—the Google Trends Dataset

Although there are many public-facing websites about infection (eg, NHS Choices [25], Bugs and Drugs on the Web [26]), these are multiple and fragmented, some focused on a single condition (eg, Bugs and Drugs) and of varying quality as these are run by patient groups, governments, and industry [27,28]. Furthermore, the search logs are not publicly available. For this reason, using search engine data for searches for infection-related terms provides a high volume and much more compelling source of public online information needs in this area over the same period of time. Therefore, to evaluate patient information needs, we used data from Google Trends [20] (Supplementary Figure 4 in Multimedia Appendix 1), which measures Google searches for particular keywords. These data measure the weekly volume of searches using a keyword, but rather than the absolute numbers of searches, a normalized value is given. This is scaled so that for a single keyword or phrase, the average value over the specified time period is 1. Therefore, a value of 2 would indicate a volume of twice the long-term average. Comparisons between terms can also be made. In this case, the normalization is done so that one term has a long-term average of 1, and the other terms have values that are scaled accordingly.

##### Google Users

Google is currently the most commonly used search engine worldwide, with 90% of the market share globally, and 80% in the United States, according to StatCounter Global Stats [29]. See Supplementary Figures 5 and 6 in Multimedia Appendix 1 for the worldwide and US data respectively. It therefore seems justifiable to use Google search data as representative of the general public’s search interests.

#### Dataset 3: Media Coverage of Infection Outbreaks—News Articles From LexisNexis

The third dataset, measuring media coverage of specific topics, is the newspaper articles retrieved from the LexisNexis database [30] (see Supplementary Figure 7 in Multimedia Appendix 1). These articles were from major world newspapers in the English language. The results can be saved as a text list, from which the dates of the articles (which are necessary for our analysis) can be extracted.

###  Analysis: Pre-Processing of NeLI/NRIC Log Data

Before Web server log data could be used, the dataset had to be cleaned. As there are site visits not motivated by specific interest that can be of high volume and at random times, it is important to try to identify and remove them from our data. The main sources of spurious accesses were *Web crawlers* and *referrer spam* (there are also accesses by the website developers during developing and testing, which were easily identifiable).

Web crawlers (also referred to as spiders) are programs that visit pages of a website, usually for the purpose of indexing the site for search engines. Web crawlers tend to visit the same sites frequently to check for updates. Crawlers can cause serious distortion of the Web log statistics, as they can produce a spike in the logs that is not due to any genuine interest in the site [31,32].

Referrer spam [33] is created by automated programs that generate Web log entries with the referrer site field set to a specific Web address. This is intended to generate free advertising if the weblogs are made available online.

In order to remove as many spurious log entries as possible, the Web logs were pre-processed with the following steps:

1. All entries with an IP address in a list of developer IPs were removed.
2. The browser type field (see Figure 4 for an example) in each log entry was examined and those that did not correspond to common Web browsers were removed. Our aim was to remove those entries that were not caused by human use of a Web browser.
3. The previous step still left a large number of entries that were clearly produced by Web crawlers. The remainder of the browser type field was examined, and any that contained certain keywords that indicate Web crawlers (specifically “bot”, “crawler”, “spider”, “slurp”, and “jeeves”) were rejected.
4. Referrer spam was removed by first finding the most common referrer websites in our logs. By concentrating on the most frequently occurring sites, the most likely referrer spam sites were identified manually. A block list of terms and site names was built up, which was used to exclude log entries during the processing phase.

### Analyzing Interest in Infection Topics

After the pre-processing that was only required for the professional needs containing NeLI/NRIC logs, the analysis of the 3 datasets was performed—each required a different technique to analyze an information need or interest in a certain infection topic.

#### NeLI/NRIC Logs

After pre-processing the logs, the remaining entries were divided into document views (ie, looking at a specific document in the library), category browses (looking at a list of documents about a specific topic, the second level of the two level taxonomy described in section 3.1.1), and searches (ie, the entry of search terms into the search box). Other accesses, such as image views or views of pages not relating to specific diseases or organisms, were not counted. We concentrated on category browsing and document views, as (1) browsing was much more commonly performed than searching (93% of the total) [34], (2) factors such as misspellings, synonyms, and complex search phrases make analysis of search terms much more complicated, and finally (3) for advanced keyword searches, an autocomplete function (which suggests keywords after the user has typed a few characters) was used. This distorts the results for searches, as the searches for partial words are also recorded in the logs.

**Figure 5:**
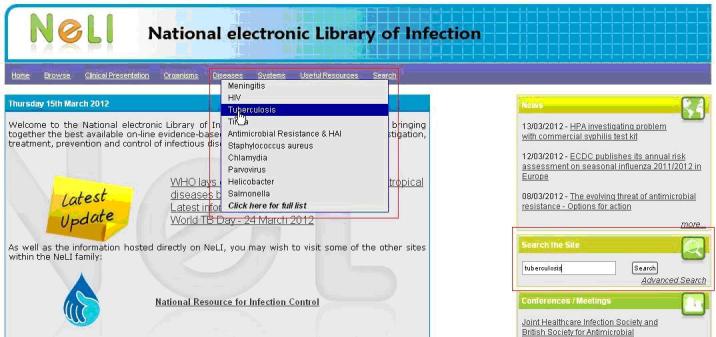
Alternative methods of finding information on NeLI using the drop down menus (browsing) and free text searching.

Finding information on NeLI/NRIC can be achieved either by using a search (either from an external search engine or by an internal search on the NeLI/NRIC site) or by visiting the site and following links or menus to navigate to the required information (see Figure 5).

After plotting graphs for professional interest in infectious diseases using the NeLI/NRIC dataset, we considered the major peaks in the graphs and attempted to identify any major policy documents that were released at the corresponding times. To verify if these documents may have caused the peak in interest, we studied the NeLI/NRIC logs to measure the download rates of the documents and matched them to the professional interest graphs (which measure the overall browsing activity for the disease).

### Public Interest: Google Trends Dataset Analysis

Public interest data were obtained by entering search terms at the Google Trends website. The data were downloaded as weekly data in comma separated value (CSV) format, using relative scaling, so that the data are scaled to make the average level over the period is 1. We chose to use single terms (eg, “norovirus”, “tuberculosis”, “*C difficile*”) instead of trying to include synonyms (“winter vomiting bug”, “TB”, “C. diff”, “superbug”, etc), partly for simplicity and partly because Google’s search algorithm can already make some allowance for synonyms and misspelled search terms. There are specific complications associated with analyzing influenza, as in addition to the common term “flu”, there are varieties of influenza that have been widely covered by the media (avian influenza, or “bird flu”, and swine influenza, or “swine flu”). As it is difficult to tell whether the results for “influenza” might have been part of a more specific query about avian or swine influenza, we found totals for “influenza” as a whole.

###  Media Coverage: Newspaper Article Analysis

Media articles were extracted from the LexisNexis database, using the same search terms as were used for the Google Trends results. The search was performed over “Major World Publications (English)” and returned articles where the keyword was mentioned near the start of the article. The similarity measure was set to “On, high similarity” to exclude duplicate articles. The articles were sorted by date and counted to give weekly totals.

Once the information from the 3 datasets was plotted as time series, we examined the correlation of the 3 signals and further investigated the real events and key publications to attempt to explain any spikes, trends, and other patterns in the data. These were gathered by searching news and press release databases from the Health Protection Agency (HPA), the European Centre for Disease Prevention and Control (ECDC), the World Health Organization (WHO) and other agencies, and in-depth analysis of access to the actual documents on NeLI/NRIC creating the peak and manually verified by infection domain experts. Also, we related the levels of professional interest with that of the public and each of these to the media coverage.

## Results

To compare the 3 datasets, we applied the same scaling that Google Trends uses to the numbers of news stories and levels of professional interest, so that the average level (or “baseline”) over the study period was 1. This means that the 3 measures could be plotted on the same scale.

Graphs were plotted showing the activity over time for NeLI/NRIC and Google Trends. Google Trends normalizes its data so that a level of 1 is the long-term average activity level over the period, and for comparison the same was done with NeLI/NRIC.

### Major Infection Outbreaks

The last decade has been eventful in the domain of infectious disease. There have been periods of emerging infections (SARS), epidemic outbreaks (avian flu), a pandemic (swine flu in 2009) as well as recurring outbreaks for common infections (influenza, MRSA). We evaluated the 3 datasets to try to understand the correlations; however, as the primary aim was to understand professionals’ needs, we investigated the NELI/NRIC dataset to determine the most accessed infection topics and disease outbreaks.

The next section describes the results of each disease separately and provides background events to illustrate the information needs of professionals and public.

### *Clostridium difficile* and MRSA

#### Introduction

*Clostridium difficile*, also written as *C difficile* or *C diff*, and MRSA are bacteria that can infect patients through cross infection, in hospitals, nursing homes, or other health care facilities, hence the commonly used term health care associated infections (HAIs). They are also linked to overuse of antibiotics causing resistance or damage to normal body bacteria, poor hygiene practice, age, and lowered immunity. Another popular term among the public is “superbugs” [35,36].

**Figure 6:**
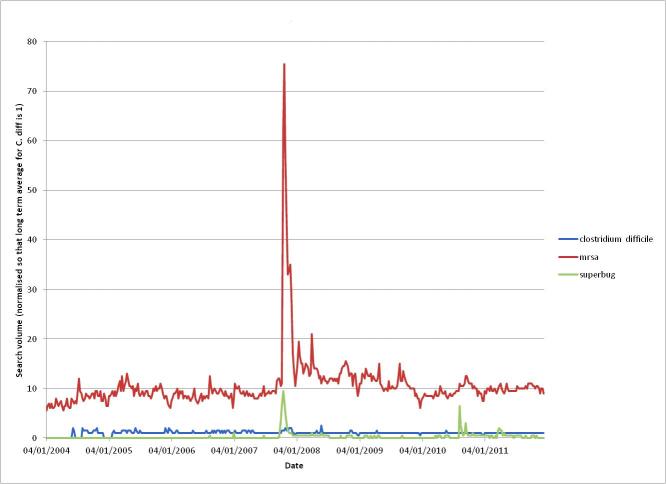
A comparison of Google searches for the terms "clostridium difficile", "MRSA", and "superbug".

Table 2 shows the number of news articles returned from searches on “*Clostridium difficile*” and “MRSA” and the articles that appear in both sets of results. There are relatively few articles (11.3%) that are about *C difficile* alone, whereas there are many more (62.3%) on MRSA alone, with about a quarter of articles (26.4%) mentioning both HAIs. Figure 6 shows a comparison of Google searches for “*Clostridium difficile*” and “MRSA” (and the term “superbug” for reference). Again, MRSA is a more popular term, with around ten times the number of searches performed. This might be partially explained by the difficulty of spelling “*Clostridium difficile*” compared to “MRSA” when searching. There is also the possibility that the public were more alarmed about MRSA, and there was a strong public support network (including MRSA action groups) bringing it to the public’s attention.

#### Results

**Figure 7:**
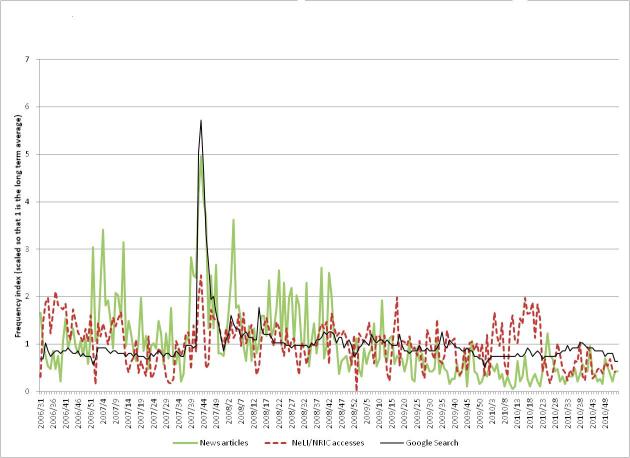
The public and professional interest, and media coverage for *Clostridium difficile* and MRSA.

Figure 7 shows the levels of (1) professional interest (measured by numbers of NeLI/NRIC accesses of the *Clostridium difficile* and MRSA taxonomy pages), (2) public interest (measured by comparative frequencies of Google searches for the terms “*Clostridium difficile*” and “MRSA”), and (3) media coverage (measured by the number of news articles mentioning “*Clostridium difficile*” or “MRSA” obtained from the LexisNexis database). Each statistic is measured weekly and normalized so that the baseline average over the period is 1.

The first observation (which applies generally to other diseases/organisms) is that the professional interest (measured by NeLI/NRIC accesses) is “noisier” (have a higher variance) than the Google Trends data. This is clearly due to Google’s far larger traffic volume. It is difficult to get exact figures for Google’s search volume, but using Google’s AdWords service indicates that in the year to January 2012, the average monthly global number of searches for the phrase “what is *C diff*” was 368,000 [37]. If other searches related to *C difficile* were to be included, the total number of relevant searches would be much higher.

#### Professional Interest

The maximum level of professional interest occurs at week 43 in 2007 (which also coincides with the maximum levels of public interest and media coverage). The professional interest level at this peak was 2.6 times the baseline level, compared to 5.0 times the baseline for media coverage, and 5.7 times for public interest. There are periods of increased activity early in the second half of 2006 and in the first half of 2010. It is likely that the increased activity in 2006 is due to the high levels of promotional activities for the newly relaunched NeLI/NRIC sites. More evidence for this comes from comparing the graphs in later sections, which show a similar pattern. The heightened activity in 2010 may be due to promotional activities or to the aftermath of pandemic flu, but this is unclear.

#### Public Interest

The public interest shows a very clear spike in 2007, coinciding with the spikes in the professional interest and media coverage. This spike is at a level that is 5.7 times the baseline. There is also a slight dip in the interest level at the end of each year, which can also be seen in the graphs for most of the other diseases. This is presumably due to lower levels of search over the period of the Christmas and New Year holidays. Also, overall public interest decreases after the 2007 spike until the end of the study period, where it is at a similar level to before 2007.

A possible interpretation of the public interest in MRSA and *C difficile* is that in 2007 it was affecting them, their relatives, and friends. In addition (in the United Kingdom at least) MRSA action groups were very active at this time and public pressure finally made the government take action, introducing targets for MRSA reduction in hospitals and nursing homes. Subsequently MRSA incidence fell and *C difficile* incidence increased, before attention turned to tackling *C difficile.*

#### Media Coverage

The media coverage also shows a clear peak in late 2007. The news stories at this time mainly focused on the findings of the Healthcare Commission in the United Kingdom concerning an outbreak of *C difficile* between April 2004 and September 2006. For example:

Scores of NHS patients were killed during Britain’s deadliest outbreak of a hospital superbug, a damning report by the government’s health watchdog reveals today. The Healthcare Commission attributed the deaths of 90 patients at the Maidstone and Tunbridge Wells hospitals in Kent to infection from *C difficile*, which causes severe diarrhoea and has taken over from MRSA as the main threat to patients.

38

This indicates that heightened media coverage in late 2007 over the Healthcare Commission report had a correlation to professionals’ needs, who were likely to access the report but around 3 times rather than 6 times more frequently than the baseline.

The article is also an example of MRSA being mentioned in a report that is mainly about *C difficile*.

**Table 2:** Number of news stories from 2006-2011 about *C difficile* and MRSA and the number of articles common to both lists.

Keyword	“*Clostridium difficile*” only	“MRSA” only	Both terms
Number of articles mentioning the term	197	464	138
Percentages	11.3	62.3	26.4

### Tuberculosis

#### Introduction

Tuberculosis (TB) is an infectious disease that is caused by a bacterium called *Mycobacterium tuberculosis*. TB primarily affects the lungs, but it can also affect organs in the central nervous system, lymphatic system, and circulatory system. Infection is spread when bacteria, coughed up by an individual with TB affecting their lungs, are released into the air and inhaled by others. TB is a major global health problem and also prevalent among people with HIV/AIDS.

#### Results

**Figure 8:**
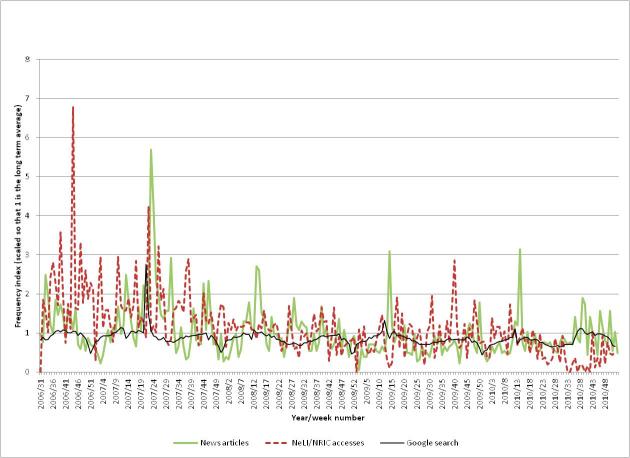
The public and professional interest, and media coverage for Tuberculosis.

From the graphs for TB (see Figure 8), it seems clear that professional and (to a lesser extent) public interest are both declining gradually. It is therefore not surprising that the NeLI/NRIC levels of interest did not show corresponding peaks or that Google Trends does not have a peak (as tuberculosis is not central to the stories, users would not use the term in searching for content).

#### Professional Interest

The overall level of professional interest appears from the graph to be declining, although this may be misleading. The graph shows the same higher level of interest for the first few months of the study period, which coincides with the promotional activities that would generate higher activity levels from professionals. When this is discounted, the remaining interest levels are more level.

A tuberculosis “Knowledge Week” was held on NeLI/NRIC in conjunction with the HPA from March 26-30, 2007, to provide health care professionals with quick and easily accessible up-to-date knowledge on the disease. This activity does not appear as a peak in the graph, as the most accessed page for the Knowledge Week was a special front page that was not counted in our analysis of searches for the disease.

**Figure 9:**
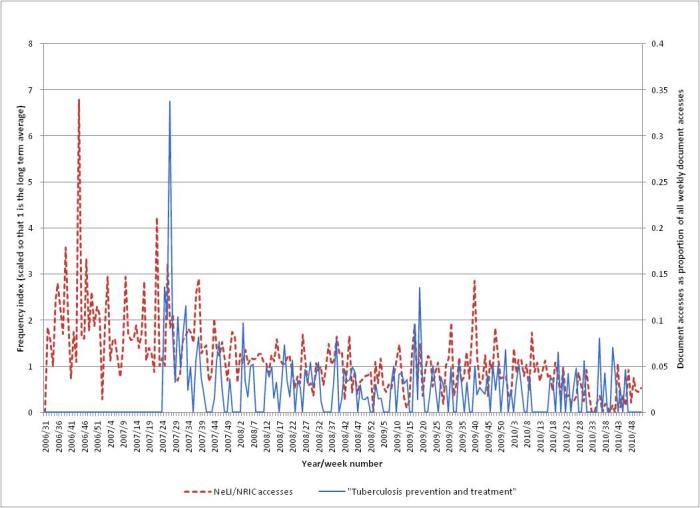
The NeLI/NRIC accesses for TB, and the accesses for the document “Tuberculosis prevention and treatment” published in June 2007.

A document “Tuberculosis prevention and treatment: a toolkit for planning, commissioning and delivering high-quality services in England” was published by the NHS on June 15, 2007, as TB was becoming a growing and expensive problem in the United Kingdom. This shows up as a peak (3.2 times baseline) in week 26 of 2007, following a public interest peak in week 21 (2.8 times baseline), a professional interest peak (4.2 times) in week 22, and a media peak (5.7 times) in week 23. Figure 9 shows the professional interest (the same NeLI/NRIC accesses as in Figure 8) for tuberculosis, together with the accesses for the document (measured as a proportion of the total weekly document accesses). Clearly there is a surge of interest in the document at the time of publication (week 26), followed by a steady decline.

#### Public Interest

The highest level in public interest occurred in May 2007 (week 21, 2.8 times baseline), which seems to be related to a story concerning a US citizen infected with TB who took a flight to Europe, potentially spreading the disease [39,40]. On this occasion, public interest was most likely triggered by the media story. The yearly dip in interest at the end of each year is also evident.

Tuberculosis is not highly prevalent in the United Kingdom but has been increasing and affects immigrants and the homeless more than other groups. This may explain some of the lack of public interest, as these affected groups may not have as much access to the Internet as other members of the public.

#### Media Coverage

There are several peaks in the media coverage, notably in mid-2007 (5.7 times baseline, mainly relating to a long-running story about a bullock kept at a Hindu temple that contracted TB [41]), mid-2008 (1.9 times baseline, mainly about a potential cull of badgers to control bovine TB [42]), and late 2008 (about a successful human windpipe transplant, which was needed due to the patient’s earlier case of TB [43]). These media stories tended to be about animal (specifically bovine) TB and a single human interest story, where the disease was incidental. Finally, there are peaks each year from 2007-2010 (2.5, 2.7, 3.1, and 3.1 times the baseline, respectively) coinciding with the World TB day, which is March 24 each year. This indicates that such events can generate media coverage.

### Meningitis

#### Introduction

Meningitis is “an infection of the meninges (the protective membranes that surround the brain and spinal cord)” and can be caused by either bacteria or viruses [44].

#### Results

**Figure 10:**
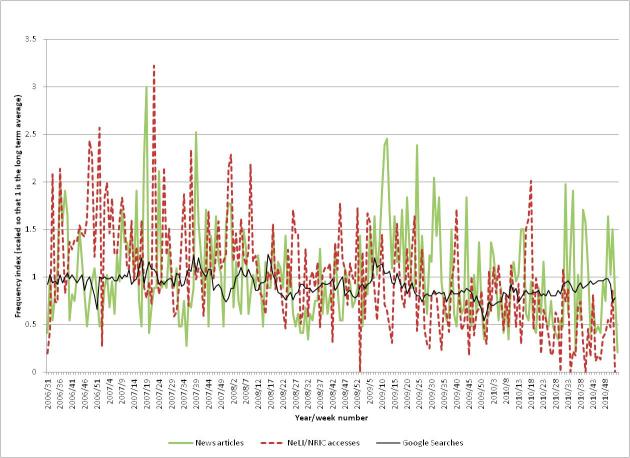
The public and professional interest, and media coverage for Meningitis.

Figure 10 shows the professional interest, public interest, and media coverage for meningitis.

#### Professional Interest

Professional interest was heightened in the years 2006 and 2007 but has declined since then. Again, some of this decline can be explained as due to enhanced levels of interest when the NeLI/NRIC sites were relaunched in 2006.

#### Public Interest

The public interest appears to be level, not deviating far from the baseline level, possibly showing a downward trend, as the graph from early 2009 is below the baseline level. The larger dips at the end of 2006 and the end of 2009 are again probably due to the holiday period. This is interesting, as it seems that the presence of heightened media coverage is not influencing the public searches.

#### Media Coverage

The peaks in media coverage show no obvious pattern. On examining the peaks and matching them to news articles, it seems that media coverage is driven by stories about individual tragedies, celebrity stories, and other human interest stories. The four largest peaks are in week 20, 2007 (3.0 times baseline), with the main focus on the singer Peter Andre who contracted meningitis [45]; in week 39, 2007 (2.5 times baseline), with stories about an individual boy’s death [46] and others about a boy who changed his accent after surgery for meningitis [47]; week 12, 2009 (2.5 times baseline), where there was no clear single focus for stories; and week 24, 2009 (2.4 times baseline), focusing on the joint suicide of the parents of a toddler who died of meningitis [48].

### Norovirus

#### Introduction

Norovirus is a seasonal disease, also called “winter vomiting disease”. “The virus, which is highly contagious, causes vomiting and diarrhoea. [...] Between 600,000 and one million people in the UK catch norovirus every year” [49].

#### Results

**Figure 11:**
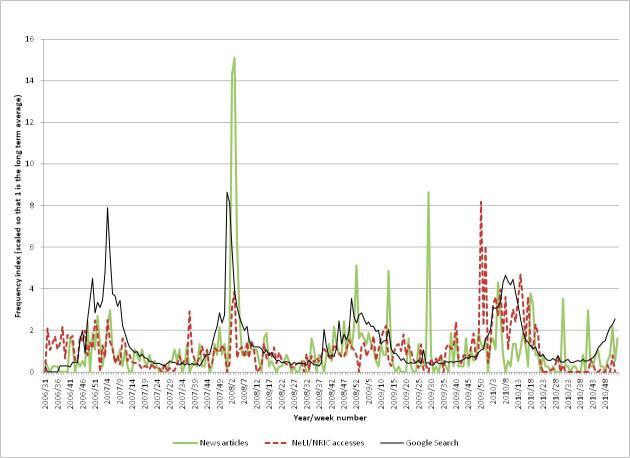
The public and professional interest, and media coverage for Norovirus.

In contrast to the other outbreaks that we have investigated in this study, there is a clear seasonal trend to the professional and public interest and media coverage (see Figure 11). Professional interest tends to mirror public interest, except for heightened activity in late 2009 and early 2010. This extra activity is probably due to publication of the HPA’s guideline “Norovirus outbreak reporting scheme” on December 14, 2009.

**Figure 12:**
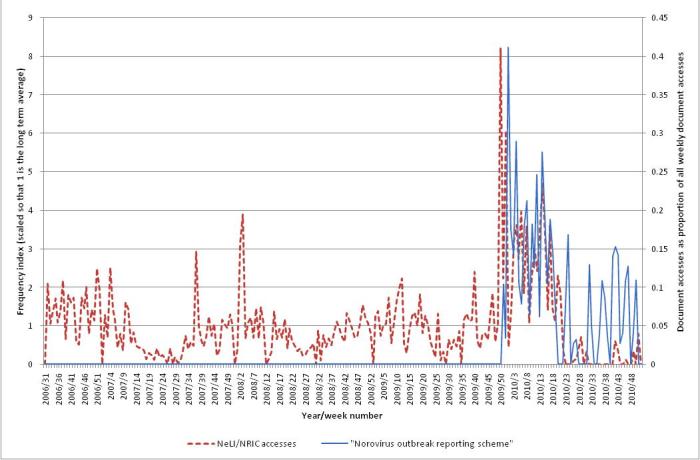
The NeLI/NRIC accesses for norovirus, and the accesses for a specific document published in December 2009.

Figure 12 shows the professional interest (the same NeLI/NRIC accesses as in Figure 11) for norovirus, together with the accesses for the document (measured as a proportion of the total weekly document accesses). There is a small peak in interest at the time of publication (the small size of this peak is due, in part at least, to the document being published near the end of that week), followed by a large peak in the next (full) week, and then a gradual decline.

#### Professional Interest

**Figure 13:**
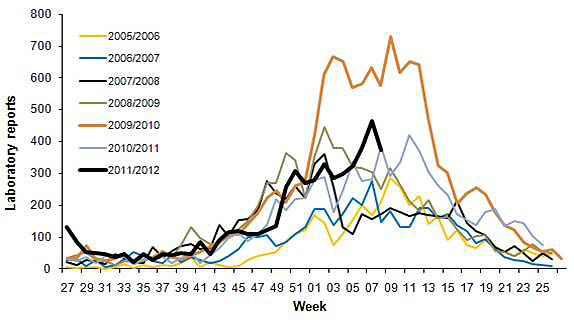
Laboratory reports of norovirus from the years 2005 to 2012 (source: Seasonal comparison of laboratory reports of norovirus (England and Wales; HPA).

Professional interest tends to mirror public interest, except for heightened activity in late 2009 and early 2010 (up to 8.2 times baseline). This extra activity is due to publication of the HPA’s guideline “Norovirus outbreak reporting scheme” in December 2009. The season 2009-2010 was also a “bad” year for norovirus outbreaks (see Figure 13).

#### Public Interest

The public interest clearly correlates to the seasonal variation in the professional interest graph and in the media coverage. The dips at the end of each year that are clearly visible in the earlier graphs do not appear here.

#### Media Coverage

There is a clear peak (15.1 times baseline) in media coverage in the winter of 2007/2008. There is also another clear spike (8.6 times baseline) at week 29 of 2009, mainly due to coverage of an outbreak of norovirus on a cruise ship [50]. The media coverage does not fit the seasonal pattern quite as well as the public and professional interest. This may be due to occasional outbreaks that can happen in summer (often on cruise ships) and also due to the media coverage including a large proportion of stories from the southern hemisphere. The professional interest levels are skewed towards the northern hemisphere, as that is where the majority of visits come from. The public interest levels are also biased towards the northern hemisphere, as the majority of Google’s traffic comes from there.

#### Comparison With Actual Disease Occurrence Data

**Figure 14:**
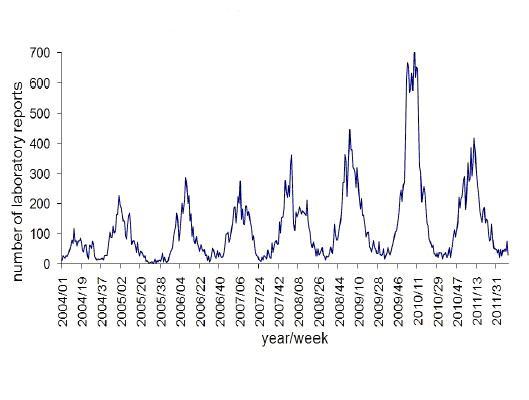
Laboratory reports of norovirus 2004-2011 (England and Wales; graph from HPA).

As the professional interest, public interest, and media coverage show such similar seasonal patterns, it is not surprising that they closely match data for the actual occurrence of the disease. Figure 14 shows the numbers of laboratory reports of norovirus in England and Wales (data from the HPA weekly epidemiological surveillance reports). As the data is from the northern hemisphere it is important to be careful in making generalizations. But there is clearly a close correspondence with the graphs of Figure 11, with the peaks occurring in the northern winter, and the troughs in the summer.

### Influenza

#### Introduction

“Influenza is a viral infection that affects mainly the nose, throat, bronchi and, occasionally, [complications occur which affect the] lungs. [...] The virus is transmitted easily from person to person via droplets and small particles produced when infected people cough or sneeze. Influenza tends to spread rapidly in seasonal epidemics.” [51]. New strains and variants of the influenza virus are constantly emerging. There are several named subsets of these, including seasonal influenza, swine influenza (“swine flu”), and avian influenza (“bird flu”).

“Seasonal flu occurs every year, usually in the winter. It’s a highly infectious disease caused by a virus. The most likely viruses that will cause flu each year are identified in advance and vaccines are then produced that closely match them.” [52]

According to an internationally accepted standard, the terms “avian influenza” and “swine influenza” refer to influenza viruses found in birds and swine, respectively [53] .However, the terms (also called “bird flu” and “swine flu”) may be used to refer to specific strains of influenza. For example, according to NHS Choices, swine flu is “the common name given to a relatively new strain of influenza (flu) that caused a flu pandemic in 2009-2010. It is also referred to as H1N1 influenza (because it is the H1N1 strain of virus)” [54].

While it may be expected that influenza would feature highest in the NeLI/NRIC accesses over the investigated period, it is actually only in sixth position. The interest in this disease on NeLI/NRIC has shown to be lower as seasonal influenza, being one of the most common diseases, does not require regular and more specialized evidence.

#### Results

**Figure 15:**
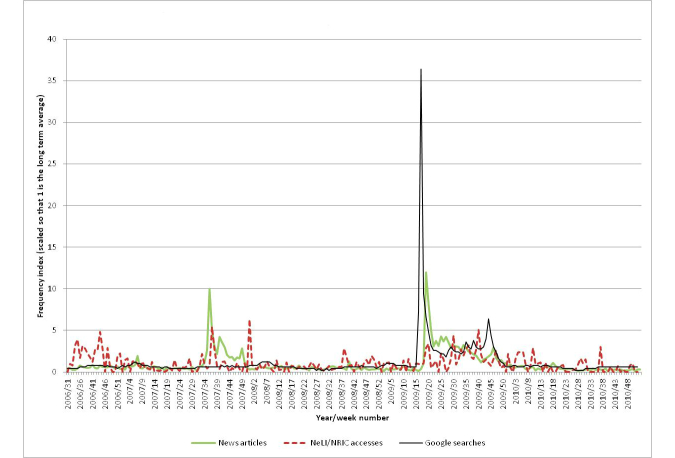
The public and professional interest, and media coverage for Influenza.

**Figure 16:**
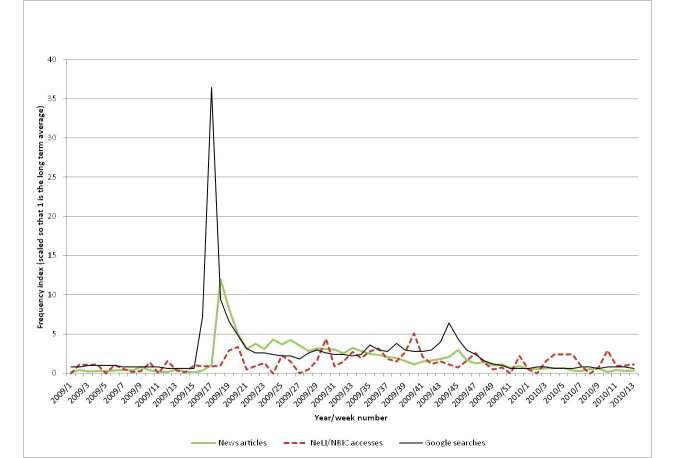
The public and professional interest, and media coverage for Influenza focusing on the period of the 2009-10 swine flu pandemic.

Figure 15 shows the professional interest, public interest, and media coverage for “influenza”. Clearly the graph is dominated by the surge of interest around the 2009 swine flu pandemic. Otherwise there was constant public interest in the disease while professionals had several spikes mostly in winter months indicating an increased information need around seasonal influenza. Apart from two isolated media interests in spring 2006 and winter 2007, there were no significant outbreaks resulting in media attention. The key exception requiring an in-depth evaluation is indeed the 2009 swine flu pandemic. Figure 16 shows just the period of 2009 and the first quarter of 2010 (the duration of the swine flu pandemic). Both the public interest and the media coverage have large peaks in the spring of 2009 (36.4 and 12.0 times the baselines, respectively), corresponding to the initial cases in Mexico and the announcement of the pandemic, but have much lower activity later when there was a second peak in flu cases (matching the findings of [17] for the media coverage). However, the public interest does show a smaller peak (6.4 times baseline) in autumn 2009.

#### Professional Interest

There is once again a heightened level of activity in the second half of 2006, presumably caused by promotional activity of NeLI/NRIC. During the H1N1 outbreak, the levels were higher than average, but there was not a large spike, as was the case for public interest and media coverage. This is probably due to the large number of competing information online resources for public and professionals that were created during the 2009 pandemic, and the public health agencies such as HPA, ECDC, and WHO held daily press conferences publishing the latest evidence and advice. For this reason, in summer 2009, NeLI/NRIC decided to add a dedicated swine flu link to their home pages to redirect visitors to the ECDC flu website for the daily updates [21].

#### Public Interest

Interestingly, public interest in the disease peaks in week 17 of 2009, while the peak in media coverage followed in week 18. However, the difference of one week is probably not significant here, as the process of collating results into weekly totals will have some uncertainties. According to a study conducted in 2009:

The highest number of articles (842) was recorded on 27 April, the day WHO raised the level of influenza pandemic alert to phase 4...There was a smaller, though still large, peak of the number of media articles on 30 April (717 articles). This appears to be linked to WHO’s announcement of pandemic alert phase 5 at 22:00 Central European Time on 29 April: many of the European media reports about this were published on 30 April. Media interest dropped considerably after 30 April.

55

April 27 is near the end of week 17 (23-29 April), and April 30 is at the start of week 18. It is interesting that the announcement of phase 6 (the pandemic level) on June 11, 2009, did not seem to generate any significant interest.

There was a second peak in public interest at week 44 (6.4 times baseline), identifying the autumn 2009 outbreak (this is discussed in the next section, where it is correlated with the media coverage).

#### Media Coverage

There is an earlier peak (10.0 times baseline) in media coverage in August 2007, which corresponds to a serious outbreak of influenza in Australia (for example [56]).

The second main peak (12.0 times baseline) occurred around the end of April 2009. This was around 6 weeks before the WHO declared that H1N1 was officially a pandemic (June 11, 2009). The heightened media interest in these weeks related to the outbreak in Mexico and speculation as to whether the disease would spread. Public interest during the autumn second peak of the disease was 5.7 times smaller than during the April peak, but is clearly visible in Figure 16, although the media did not give the topic much attention at this crucial time.

## Discussion

### Principal Results

In this paper we have analyzed the information needs of public and professionals around key infectious disease outbreaks and events in the 4.5 years from the end of July 2006, until the end of 2010. We compared these with media coverage to illustrate where the media interest could have fueled public interest in the disease and what the reaction was of professionals to key outbreaks and policy changes. Based on the results, the diseases fall into 4 groups:

- MRSA and *Clostridium difficile*: High prevalence, reducing rapidly with new government targets and emphasis on surveillance/reporting;
- Tuberculosis and meningitis: low prevalence;
- Norovirus: seasonal; and
- Influenza: 2009 mass media attention and pandemic event.

The next sections will discuss the results in more detail.

We found that a triangulation of (1) longitudinal Web log data from the NeLI/NRIC infection portals to evaluate the professionals’ needs around infection as a primary goal, (2) Google Trends in these topics to find a complementary public interest, and (3) media coverage from LexisNexis provides the desired correlation to answer our research questions listed in section 3:

1. How do health care professionals’ online search needs around infection differ from public needs?
2. Does media coverage contribute to the information needs for infection events among public?
3. How are incidents of a disease and major policy events related to information needs of professionals?

Our findings include (corresponding research questions 1-3 are listed in parentheses after each finding):

1. We found that public needs in infection are much more static and do not relate to disease occurrence and media coverage as much as professionals whose needs inevitably increase with a public health event or a key policy change. (for all diseases examined except influenza discussed below) (RQ1).
2. However, for events of major media interest, such as MRSA/*C difficile*, media coverage resulted in a major public interest (such as the late 2007-early 2008 UK outbreak). (RQ2).
3. Meningitis was a clear example of a disease that has a heightened media coverage that tends to focus on individual tragic cases and celebrity stories (RQ2).
4. Professionals’ interest did not follow media coverage, but spikes in interest occurred during outbreaks (MRSA, *C difficile*) release of major national policy or important document (for example, the Healthcare Commission report on *C difficile* “Investigation into outbreaks of *C difficile* at Maidstone and Tunbridge Wells NHS Trust” and the HPA document on norovirus in 2009 “Norovirus outbreak reporting scheme”) (RQ1).
5. An exception was norovirus, which showed a seasonal pattern for both groups and matched the periodic disease occurrence (RQ3).
6. Influenza was of a major concern during the H1N1 outbreak in 2009, creating massive information needs among the public. Also in autumn 2009, the public interest again peaked, but on a smaller scale and also irrespective of the media coverage. However, the media coverage was on a large scale around June 2009 when WHO officially declared the H1N1 outbreak to be a “phase 5” pandemic (RQ3).
7. Additional results (not corresponding to original research questions RQ1-3): The professional interest was heightened early in the study period for all diseases. This appears to be due to the promotional activities that surrounded the relaunch of NeLI/NRIC in 2006. The professional interest reverted to a more even level after a few months. This is also reflected in the overall graph of traffic for NeLI/NRIC (see Figure 4). Finally, public interest is often difficult to quantify due to the plain text nature of searches and the fact that slang terms are often used (for example “superbug”, Figure 6).

In general, we concluded that media plays a role in influencing public information needs but is not as crucial as is often assumed. Professionals naturally respond to disease occurrence, events, or publication of key documents or policy changes that drive their information needs.

### Limitations

Studying information online needs is very difficult, and research seems to pay little attention to uncontrolled study and analysis of Web server logs for professional and public information needs. Due to the nature of the data available, we have had to make a number of assumptions in this study:

- We have assumed that a majority of NeLI/NRIC users are health care professionals, compared to Google searches. So we therefore assume that NeLI/NRIC accesses better reflect the interests of health care professionals, whereas Google searches reflect the interests of the wider public. We claim that this assumption is reasonable, as (1) the NeLI/NRIC websites are designed to provide specialist information targeted and promoted at infection professionals, which would be of less interest to the general user, and (2) there are many websites that are more accessible to the general user (such as NHS Choices [25]).
- We have assumed that the number of newspaper articles found via LexisNexis mentioning a keyword near the start of the article is a suitable measure of media coverage. More complex measures could be used, perhaps taking into account the number of words in the article or the readership of the newspapers. Furthermore, other media could be considered, such as television and radio, or social media, such as Twitter.
- We assumed that levels of keyword searches were sufficient to measure interest in particular topics. For NeLI/NRIC, we measured the accesses of a particular topic page, whereas for media coverage and Google searches, we used specific keywords (due to the nature of the available data). In these cases, we are ignoring possible misspellings and synonyms that would have reflected interest in the topic.
- For commercial reasons, Google does not release details of how its search engine algorithms work, and so it is difficult to determine exactly what the Google Trends data represents (whether it includes misspellings and synonyms, for example).
- There are many limitations to using Web server logs to analyze user behavior: (1) it is not possible to resolve IP addresses to individual users as one IP address can represent many users, (2) despite all the efforts discussed we’ve discussed, it is not possible to identify all non-human users, eg, spiders and crawlers, and importantly (3) Web logs do not provide any insight into *why* users did what they did on the site and whether they were or were not dissatisfied with the results [21].

### Conclusions

In the last two decades, the Internet has revolutionized the way we seek up-to-date evidence and information for public, in particular, during major infection events and outbreaks. Also, the role of online media with increasing coverage of public health events has contributed to the demand for information. In this study, we compared professional and public online information needs around major infection events and outbreaks over the period from mid-2006 to the end of 2010, as well as relevant media coverage.

We investigated in depth six diseases with the highest online traffic on NeLI/NRIC: *Clostridium difficile*, MRSA, tuberculosis, meningitis, norovirus, and influenza. The results illustrated that public information needs remain steady and do not necessarily follow media coverage unless the event is widely covered (MRSA/*C difficile* and influenza).

As expected, professionals’ interest did not follow media coverage but spikes in interest occurred during major outbreaks (MRSA and *C difficile*) and around the release of major national policy or other important documents (eg, the Healthcare Commission’s report on *C difficile*, entitled “Investigation into outbreaks of *Clostridium difficile* at Maidstone and Tunbridge Wells NHS Trust”) and the HPA document on norovirus in 2009 (“Norovirus outbreak reporting scheme”). The exception was norovirus showing a seasonal pattern for both groups and matching the periodic disease occurrence. Influenza was of a major concern during the H1N1 outbreak in 2009 creating massive information needs among the public in the spring in line with the media coverage and again in the autumn of 2009, this time regardless of the media coverage.

Therefore, public health agencies with responsibility for risk communication of public health events, in particular during outbreaks and emergencies, need to collaborate with media in order to ensure the coverage is of the highest quality and evidence-based while professionals information needs remain mainly fulfilled by online open access to key resources.

## Multimedia Appendix 1

Supplementary figures.

## Abbreviations

*C difficile*: *Clostridium difficile*
CeRC: City eHealth Research Centre
ECDC: European Centre for Disease Prevention and Control
EU: European Union
HAI: health care associated infections
HPA: Health Protection Agency
MMR: measles, mumps, and rubella
MRSA: multi-resistant Staphylococcus aureus
NeLI: National electronic Library of Infection
NHS: National Health Service (UK)
NRIC: National Resource for Infection Control
SARS: Severe Acute Respiratory Syndrome
TB: tuberculosis
WHO: World Health Organization
