# Supplementary material for: Major Infection Events Over 5 Years: How Is Media Coverage Influencing Online Information Needs of Health Care Professionals and the Public?
Source: J Med Internet Res. 2013 Jul 15;15(7):e107. doi: 10.2196/jmir.2146 (PMC3713905; doi:10.2196/jmir.2146)
Supplement: Supplementary file 1 [file jmir_v15i7e107_app1.pdf]

Multimedia Appendix 1. Supplementary figures.

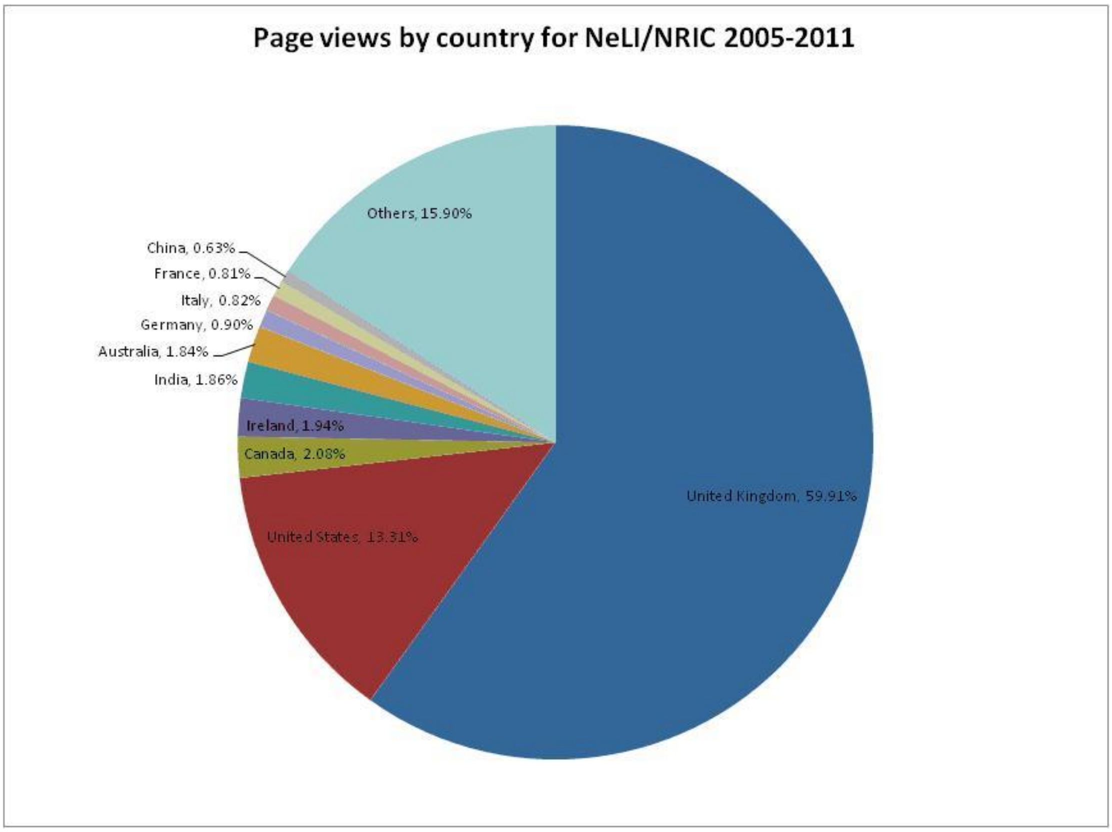

Figure 1: The combined proportion of visitors to NeLI and NRIC by location.

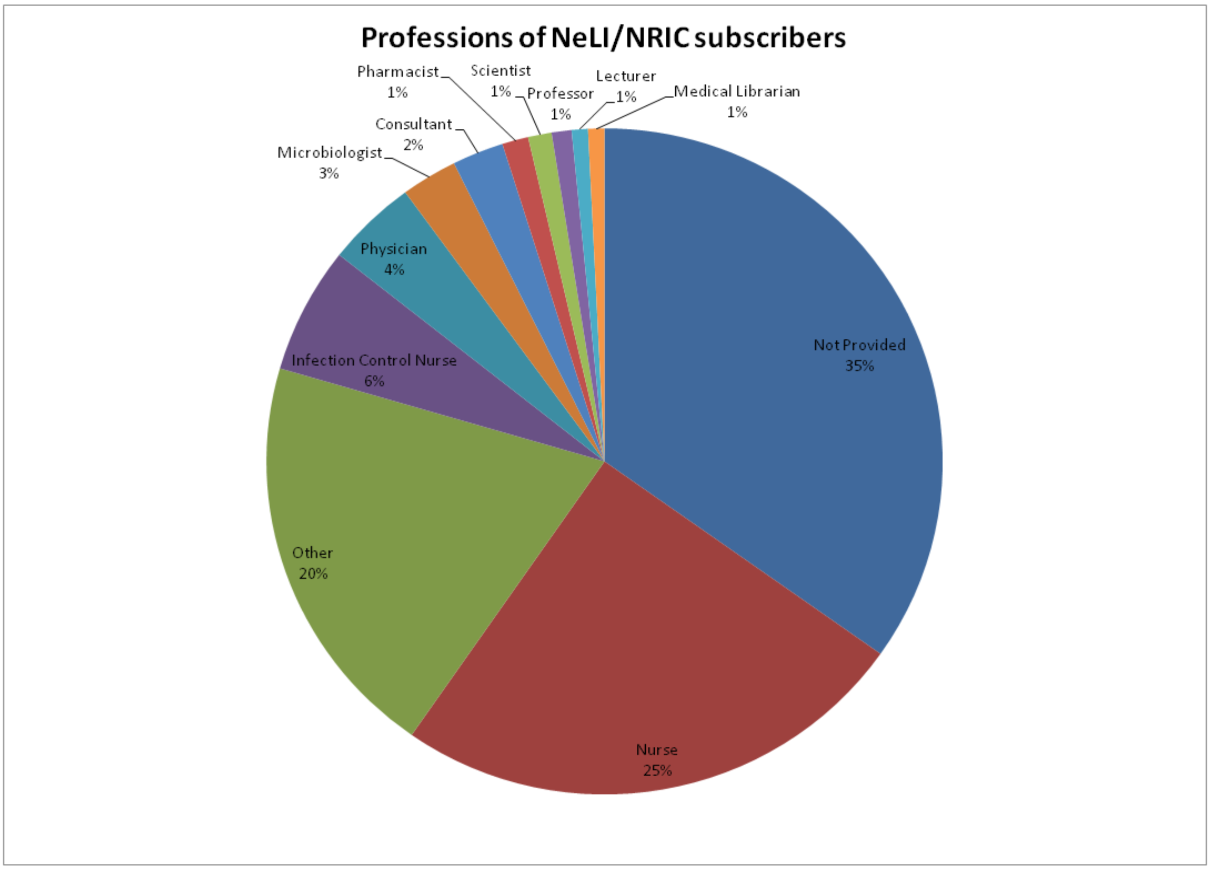

Figure 2: Details of professions provided by NeLI/NRIC subscribers.

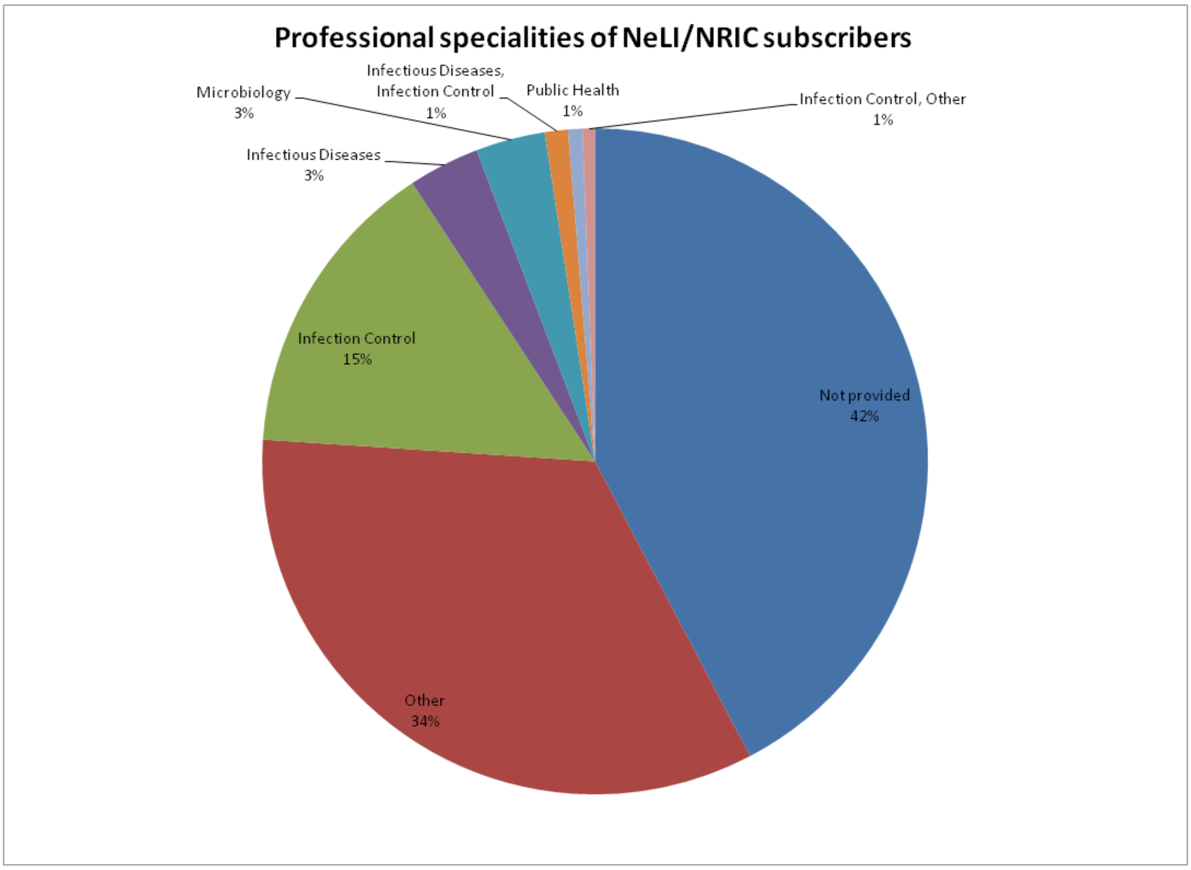

Figure 3: Details of professional specialities provided by NeLI/NRIC subscribers.

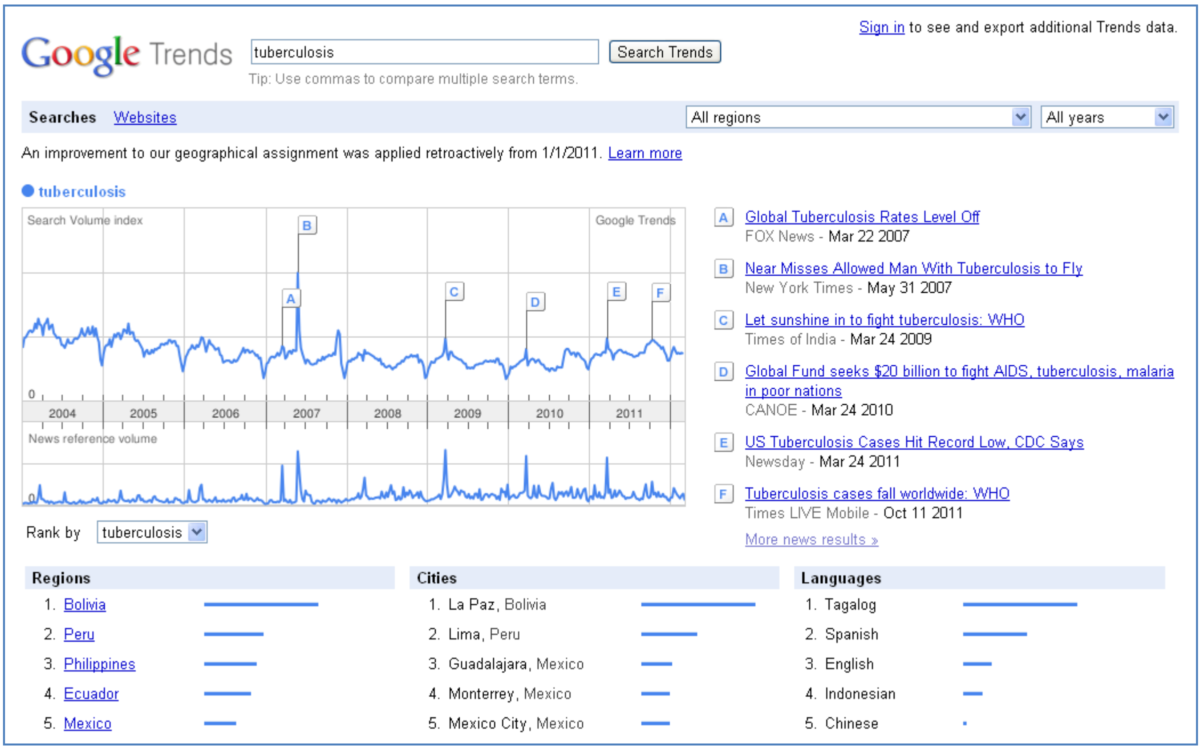

Figure 4: The Google Trends interface, showing results for the search term "tuberculosis".

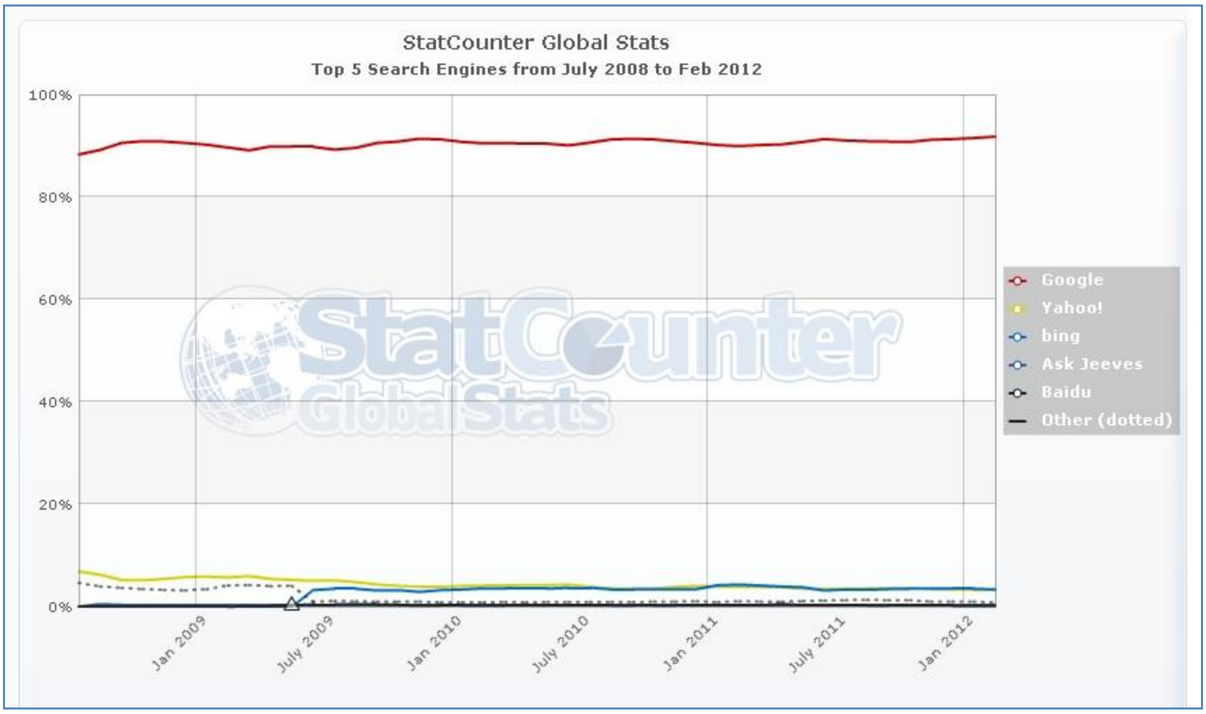

Figure 5: The monthly worldwide market share of search engines, according to StatCounter Global Stats (http://gs.statcounter.com/).

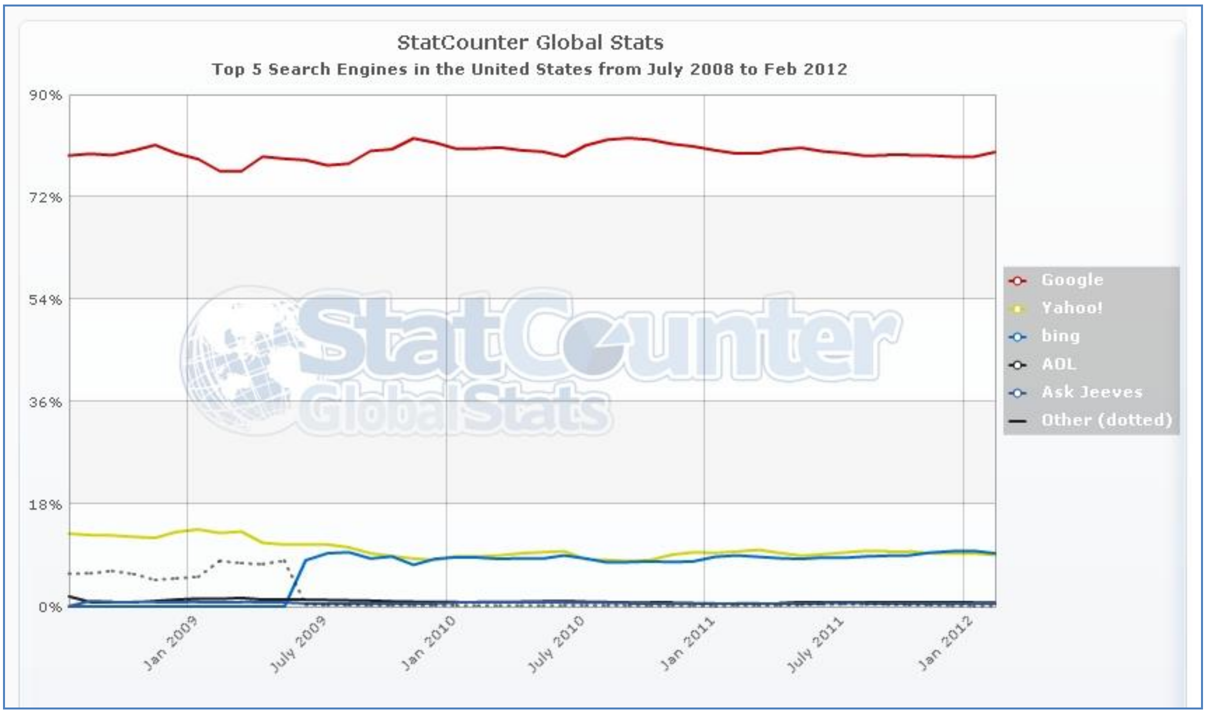

Figure 6: The monthly US market share of search engines, according to StatCounter Global Stats (http://gs.statcounter.com/).

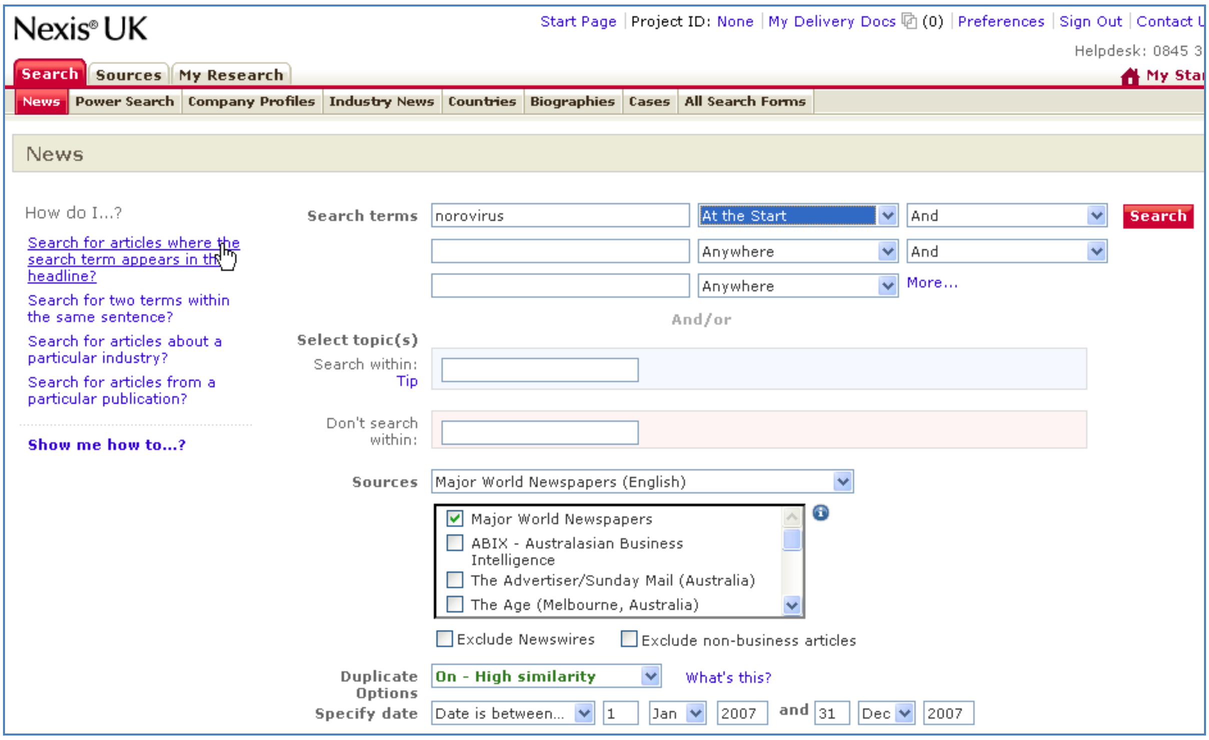

Figure 7: The LexisNexis search interface, showing a typical search for articles about the topic “norovirus”.
